# Mapping of Urinary Volatile Organic Compounds by a Rapid Analytical Method Using Gas Chromatography Coupled to Ion Mobility Spectrometry (GC–IMS)

**DOI:** 10.3390/metabo12111072

**Published:** 2022-11-05

**Authors:** Giulia Riccio, Silvia Baroni, Andrea Urbani, Viviana Greco

**Affiliations:** 1 Department of Basic Biotechnological Sciences, Intensivological and Perioperative Clinics, Università Cattolica del Sacro Cuore, 00168 Rome, Italy; 2 Department of Diagnostic and Laboratory Medicine, Unity of Chemistry, Biochemistry and Clinical Molecular Biology, Fondazione Policlinico Universitario A. Gemelli IRCCS, 00168 Rome, Italy

**Keywords:** GC–IMS, metabolomics, urine, volatile organic compounds, volatilomics

## Abstract

Volatile organic compounds (VOCs) are a differentiated class of molecules, continuously generated in the human body and released as products of metabolic pathways. Their concentrations vary depending on pathophysiological conditions. They are detectable in a wide variety of biological samples, such as exhaled breath, faeces, and urine. In particular, urine represents an easily accessible specimen widely used in clinics. The most used techniques for VOCs detections are expensive and time-consuming, thus not allowing for rapid clinical analysis. In this perspective, the aim of this study is a comprehensive characterisation of the urine volatilome by the development of an alternative rapid analytical method. Briefly, 115 urine samples are collected; sample treatment is not needed. VOCs are detected in the urine headspace using gas chromatography coupled to ion mobility spectrometry (GC–IMS) by an extremely fast analysis (10 min). The method is analytically validated; the analysis is sensitive and robust with results comparable to those reported with other techniques. Twenty-three molecules are identified, including ketones, aldehydes, alcohols, and sulphur compounds, whose concentration is altered in several pathological states such as cancer and metabolic disorders. Therefore, it opens new perspectives for fast diagnosis and screening, showing great potential for clinical applications.

## 1. Introduction

Volatilomics is a recent and promising branch of metabolomics that focuses on the study of small molecules and volatile organic compounds (VOCs) with significant potential for biomarker discovery and screening [1].

Specifically, VOCs are a large and highly differentiated class of molecules, continuously produced in the human body and released as intermediates or products of cellular metabolic pathways. They include ketones, aldehydes, alcohols, sulphur compounds, esters, aromatic hydrocarbons, and terpenes, whose concentrations vary depending on pathophysiological conditions, and are detectable in a wide variety of biological samples (exhaled breath, urine, blood, faeces, and skin).

In recent years, the diagnostic potential of VOCs has been strongly recognised. There is an increasingly evident correlation between the profile of VOCs and various diseases, including diabetes [2], irritable bowel syndrome, asthma [3], and, above all, cancer [4].

Compared to other types of metabolites, which have to be extracted from tissues or body fluids prior to analysis, VOCs are directly accessible in the gas phase (headspace), thus requiring minimal sample preparation and enabling noninvasive, real-time monitoring.

Consequently, headspace analyses may find easy applicability in the clinical setting.

As for the biological matrix, in addition to breath, urine is the most used fluid for the detection of VOCs. It is a biological fluid easy to collect with a noninvasive sampling, less complex than other fluids [5], and available in large volumes so VOCs can be detected even in high concentrations.

Therefore, it represents a well-suited source for VOCs metabolomics investigation. 

Moreover, urinary VOCs can vary both in concentration and in the types of molecules depending on several variables such as diet, therapies, genetic factors, and smoking habits, which must be taken into account during analysis [6]. 

Gas chromatography coupled to mass spectrometry (GC–MS) is the gold standard technique used to detect urinary VOCs. GC–MS is an extremely useful tool; however, it is also extremely expensive and time-consuming, and it requires highly skilled personnel and is not portable. Therefore, it is not a suitable technique to be implemented in the clinical setting [1,6,7].

As a result, there is an urgent need for fast and non-invasive innovative methodologies for VOCs analysis that can be implemented in clinical early diagnosis applications. 

In this context, the aim of this study is to develop an alternative analytical method using a high-sensitivity gas chromatographic system coupled to an ion mobility spectrometer (GC–IMS) for the rapid detection of urinary VOCs. 

To the best of our knowledge, GC–IMS has already been applied to detect different VOCs profiles in breath samples and to distinguish between diagnostic groups related to inflammatory bowel disease (IBD) [8]. Furthermore, IMS is finding great application in the analysis of exhaled breath samples of lung cancer patients [9]. Recently, the potential of VOCs profiling in the urine of lung cancer patients to differentiate them from healthy subjects is also being evaluated with GC–IMS and an electronic nose (e-nose) [10]. The main advantages of this technology were highlighted, including non-invasiveness, portability, ease of use, and cost-effectiveness. 

The implementation of this method could open up new perspectives for extremely rapid diagnosis and screening, showing great potential for clinical applications.

## 2. Materials and Methods

### 2.1. Chemicals and Materials

The ketone mix was composed of six ketones (2-butanone, 2-pentanone, 2-hexanone, 2-heptanone, 2-octanone, and 2-nonanone) (S.C.A.T. Europe GmbH, Walldorf, Germany). Chemical standards, such as 4-heptanone, were of analytical grade (Thermo Fisher Scientific, Waltham, MA, USA). In addition, 20 mL headspace vials (screw top, rounded bottom, clear glass vial (vial size: 22.5 × 75.5 mm)) and caps (screw cap 18 mm, argent magnetic, PTFE/silicone septum, septum thickness 1.5 mm) were sterile (Thermo Fisher Scientific, Waltham, MA, USA). Needles (calibre 21 G, colour green, size: 0.8 × 50 mm) were purchased from Agani Needle (Terumo Europe N. V., Leuven, Belgium) and a 5 mL Luer Lock Solo syringe was purchased from Injekt B. Braun (B. Braun, Melsungen, Germany). MilliQ water was prepared using the Elix^®^ 70 water purification system (Merk, Dramstadt, Germany).

### 2.2. Analytical Method Validation

For column normalisation and internal calibration, a standard mixture of six ketones (S_0_ as defined in Table 1) was analysed. It included 2-butanone, 2-pentanone, 2-hexanone, 2-heptanone, 2-octanone, and 2-nonanone (mixed volume ratio 1:1:1:1:1:1). Seven different solutions (M_1_, M_2_, M_3_, M_4_, M_5_, M_6_, and M_7_) were prepared at the concentrations outlined in Table 1. An amount of 2 mL of each solution was put in a screw vial and left to settle for 10 min to allow the transition of VOCs to the gas phase in the headspace. Then, 3 mL of vial headspace was withdrawn and injected in the instrument. Each measurement was performed in triplicate after the blank in the experimental condition.

### 2.3. Sample Collection

Urine samples were collected at Clinical Chemistry, Biochemistry, and Molecular Biology Operations Unit (UOC), Fondazione Policlinico Universitario A. Gemelli IRCCS (Rome, Italy). All the investigations were performed on the residual sample aliquots after the conclusions of all clinical procedures. Samples were stored at room temperature for no more than six hours in order to avoid the degradation. The pH of urine samples was in the range of 5.0–7.5.

### 2.4. Sample Preparation

An amount of 2 mL of urine sample was withdrawn from the residual urine and immediately put in 20 mL glass screw vials. Vials were closed with the appropriate screw cap equipped by a Silicon/PTFE septum to allow for picking the gas phase from the headspace. Samples were incubated at 37 °C for 10 min before the analysis, facilitating the transition and the stabilisation of VOCs between the liquid phase and vial headspace. An amount of 3 mL of headspace air was withdrawn with a sterile syringe from the vial and injected through a Luer adapter into the system. Samples were directly injected without any pre-concentration or extraction.

### 2.5. GC–IMS Analysis

Samples were analysed by a GC–IMS system (G.A.S., Dortmund, Germany), a combination of a gas chromatograph and an ion mobility mass spectrometer. Volatile chemical compounds, which are contained in the vial headspace, are physically pre-separated by GC and detected by IMS after a second separation in a drift tube, allowing for analysis of complex mixtures with the concentration at the parts per billion level (ppb/μg/L). Technical features are shown in Table 2. Briefly, GC–IMS is equipped with a gas recycling flow unit (CGFU) to purify ambient air, used as a carrier gas at 40 °C in GC and as a drift gas at 45 °C in IMS. The flow rate of carrier gas is set at 5 mL for the first 30 s and increased to 30 mL/min within 10 min, while the drift gas flow rate is set at 150 mL/min. A capillary DB wax column, thermostated at 40 °C, is used. VOCs ionise through a β-radiation tritium (^3^H) source with 300 MBq of activity in positive ion mode. After a soft chemical-ionisation, ions move to a 10 cm drift tube driven by a ±5000 V electric field. Drift gas molecules enter in the drift tube and collide with analytes accelerated by the electric field, whose separation depends on the molecular weight, charge, and spatial structure. They reach a Faraday plate where the ion current is measured as a function of time. The overall time of analysis is 10 min.

### 2.6. Data Analysis

Spectrum visualisation, organisation of data measurement, and setting of experimental conditions were enabled by VOCal software (v0.1.3, G.A.S., Dortmund, Germany). Column normalisation was carried out by analysing the standard mixture of six ketones with increasing molecular weight and retention indexes (Ri), or Kovats indexes were calculated by an algorithm of libraries of the software VOCal based on the formula:

$$I=100\times \left[n+\left(N-n\right)\frac{\log\left(Rt_{unknown}\right)-\log\left(Rt_{n}\right)}{\log\left(Rt_{N}\right)-\log\left(Rt_{n}\right)}\right]$$

where the variables are as follows:

*I*, the Kovats retention index of the peak;
*n*, the carbon number of the shorter alkane;
*N*, the carbon number of the longer alkane;
*Rt*, the retention time registered.

The retention time, Rt, and the drift time, Dt, are the two main values recognised by the device. In particular, Rt is defined as the time in seconds that a compound spends in the column after being injected. Dt is the time an ionised compound takes to reach the detector during an acceleration due to an electric field in a drift tube. The spectra obtained are a three-dimensional pseudo-colour representation reporting the Rt on the y-axis and Dt on the x-axis.

After all acquisitions, the areas of the most relevant peaks are highlighted and selected using the VOCal software. The identification of VOC species is based on the Ri and Dt of each peak calculated from those of standard ketones using the IMS database of GC/IMS Library Search tool software (NIST2014 db wax).

Calibration was performed by analysing the ketone mix at seven different concentrations. Afterwards, the quantification was carried out for the ketone mix compounds as well as for urine samples.

## 3. Results

### 3.1. Analytical Method Validation

**Figure 1:**
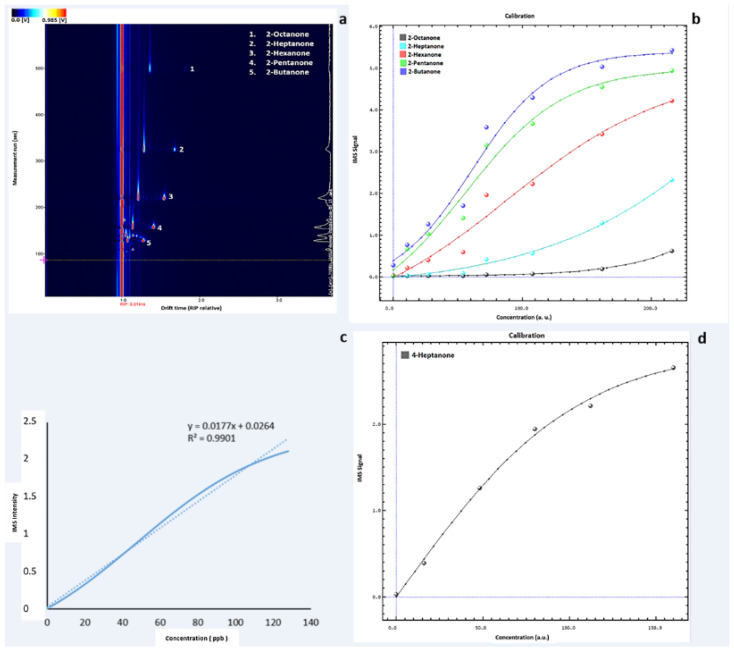
(**a**) Example of GC–IMS output of the ketone mix profile at the concentration of 108 ppb. The detected compounds have been highlighted. Each compound’s Dt has been normalised by means of the software application to the signal of the reaction ion peak (RIP). It represents the total number of ions available for ionisation, and therefore, it is used as the reference signal. The colour representation corresponds to a three-dimensional spectrum. An increasing concentration of VOCs is outlined by the colour change from blue to red. (**b**) Calibration curve obtained with the VOCal software by measuring the ketones mixture at seven different concentrations in the range of 218.4–10.8 ppb. Each colour corresponds to a detected compound in the ketone mix (blue = 2-butanone; green = 2-pentanone, red = 2-hexanone; light blue = 2-heptanone; black = 2-octanone). Dots represent the signal intensity for the concentrations analysed (expressed as arbitrary unit, a.u.); lines show the fit of the calibration curve. (**c**) Linearity range for 4-heptanone analysed by means of GC–IMS. The linearity curve and the regression line are reported for the concentration range of 0–128 ppb. (**d**) Calibration curve obtained with the VOCal software by measuring 4-heptanone at five different concentrations in the range of 0–160 ppb.

Before the analysis of biological samples, an analytical validation of instrumental parameters is carried out. First, in order to identify VOCs, column normalisation is carried out by analysing a mixture of ketones including compounds with different molecular weights (2-butanone, 2-pentanone, 2-hexanone, 2-heptanone, 2-octanone, and 2-nonanone). Their Rt and Dt cover a range of our interest, in which most of the common volatile compounds contained in the human biological samples are included and detectable with this device. A typical spectrum of the ketone mix at the concentration of 108 ppb is shown in Figure 1 (Figure 1a).

As reported in the method section (Table 1), seven solutions of the ketones mixture at different concentrations are analysed in triplicate to obtain a calibration curve. The 2-nonanone signal is extremely low; thus, it is not shown (Figure 1b).

In particular, 4-heptanone is selected to assess the linearity range. The standard solutions of this VOC at different concentrations (8, 16, 48, 80, 112, and 160 ppb) are analysed. Specifically, the curve for 4-heptanone is linear and statistically acceptable (R^2^ ≥ 0.9901) in the concentration range of 0–128 ppb, while for higher concentrations (>128 ppb), the linearity is slightly lower (R^2^ ≥ 0.9802) (Figure 1c).

To assess the sensitivity of the method, LOD is calculated from the regression slope. The value found from the regression slope is 4.66 ppb, which is close to the experimentally detectable LOD value by analysing 4-heptanone solutions at low concentration in the range of 1–5.5 ppb as reported by the instrumental features (Figure 1d).

### 3.2. VOCs Analysis in Urine Samples

In order to obtain a comprehensive urinary VOCs profiling, 115 urine samples are analysed by the GC–IMS device as described in the methods section. Our test does not require any sample treatment, thus greatly reducing the analysis time. Samples are directly injected and analysed by GC–IMS.

**Figure 2:**
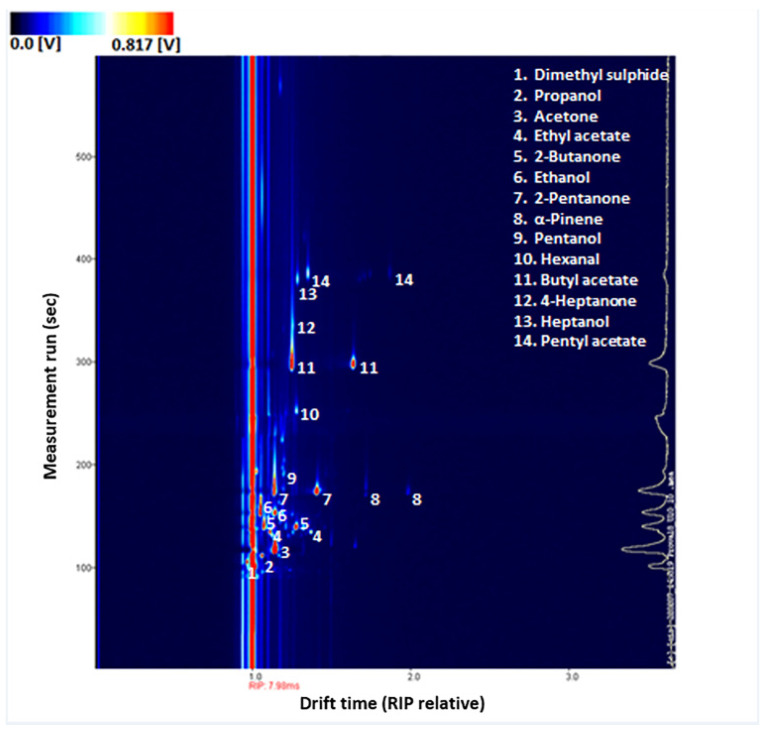
Example of GC–IMS spectrum of a urine sample. Detected VOCs have been highlighted. Increasing concentrations of VOCs are outlined by the colour change from blue to red.

For each sample, a three-dimensional GC–IMS spectrum is obtained (Figure 2). Seven main classes of volatile compounds are identified as reported in Table 3. These include ketones, sulphur compounds, esters, aldehydes, alcohols, and aromatic hydrocarbons, terpenes.

**Figure 3:**
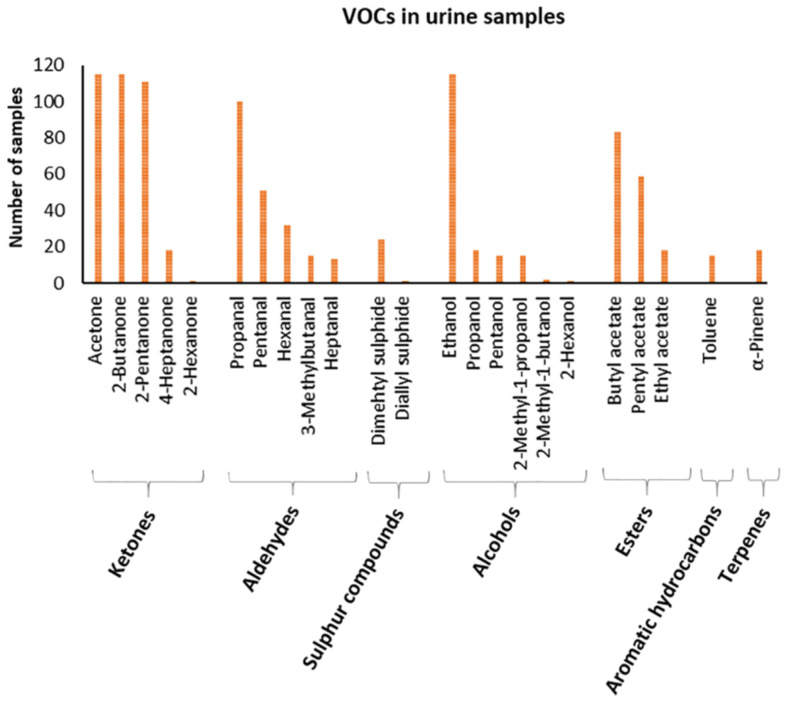
Graphical representation of VOCs profile in urine samples. The x-axis shows detected VOCs grouped in classes of molecules, and the y-axis shows the number of samples showing that VOC.

The molecules identified occur heterogeneously within the population (Figure 3).

In particular, ketones represent the main compounds. Among these, acetone and 2-butanone are detected in the entire sample population. Then, 2-pentanone is found in 97% of the population, 4-heptanone is detected in 16% of the population, and finally 2-hexanone is found in only one sample (0.87%).

Among the aldehydes class, propanal is found in 87% of the population, pentanal is found in 44%, hexanal in 28%, 3-methyl butanal in 13%, and heptanal in 11%.

Sulphur compounds, such as dimethyl sulphide and diallyl sulphide, are found in 21 and 0.87%, respectively.

The class of alcohols is the most abundant in number of detected compounds: ethanol is present in the entire population, propanol in 16% of the population, pentanol in 13%, 2-methyl-1-propanol in 13%, 2-methyl-1-butanol in 1.74%, and 2-hexanol in 0.87%.

Regarding the class of esters, butyl acetate is the most abundant and is found in 72% of the population, pentyl acetate is found in 51%, and ethyl acetate in 16%.

Among the aromatic hydrocarbons, toluene is found in 13% of the population and, among the terpenes, α-pinene is found in 16%.

### 3.3. VOCs Identification in a Sub-Population of Urine Samples

The presence of some exclusive VOCs is related to a specific subpopulation of urinary samples. This group includes 15 samples characterised by a value of ketone bodies higher than 60 mg/dL. We dwell on their analysis.

Specifically, six classes of VOCs are identified. Among these, most overlap those identified in all other samples. However, some specifically distinguish these samples, including 2-hexanone, 3-methylbutanal, pentanol, 2-methyl-1-propanol, and 2-hexanol. In particular, 3-methylbutanal (aldehydes class), pentanol, and 2-methyl-1-propanol (alcohol group) are detected in all the subpopulation. Some details on the possible origin of the detected VOCs are reported in Table 4 [11].

## 4. Discussion

To the best of our knowledge, to date, the most commonly used sampling procedures for VOCs analysis are Solid-Phase Micro Extraction (SPME) for the headspace and Stir Bar Sorptive Extraction (SBSE), N,O-Bis (trimethylsilyl)trifluoroacetamide (BSTFA) derivatisation, or centrifugation for the liquid phase [12,13]. These are followed by metabolomics analysis based on GC–MS, High-Performance Liquid Chromatography with an Electrospray Ionisation source and a Time-of-Flight Mass Spectrometry detector (HPLC–ESI–TOF), Selected-Ion Flow-Tube Mass Spectrometry (SIFT-MS), and sensors (e.g., Electronic Nose, e-Nose) [14,15]. Although these are considered the gold-standard techniques for urinary VOCs detection, they are extremely expensive and time-consuming and are, thus, not suitable for fast clinical applications. In this perspective, we develop and validate an innovative analytical method to overcome some of the limits reported so far. The main strengths of our method are its ease of use and rapid results. In particular, our analysis is performed on a GC–IMS. Both the dual-physical separation of VOCs and the high sensitivity of the IMS allow identification of compounds at the ppb level. In parallel, we use a simple device, which allows for the direct introduction of the sample in the equipment, avoiding the alteration of the analytes concentration due to extraction or pre-concentration methods. This method provides results in 10 min. The extremely low time and cost of analysis make it a particularly useful technique for fast initial screening.

Based on our results, a good level of sensitivity is achieved and a linearity range is supplied at the concentration of interest (from 5 to 130 ppb).

In order to obtain a comprehensive and fast mapping of urine volatilome, this method is applied to a first cohort of 115 urine samples from a heterogeneous population of patients without a specific preselection. Twenty-three VOCs related to seven different classes of molecules are detected. As shown in Table 3, their origin can be diverse, including endogenous synthesis and/or production resulting from microbial metabolism and external sources [11]. Ketones are one of the major classes of molecules detected in urine samples. As reported [16], they are common in urine of both healthy and ill subjects. In addition, acetone, 2-butanone, 2-pentanone, and 4-heptanone are the major ketones detected in our samples. Acetone is present in all samples, and it is the most abundant VOCs. This endogenous compound can derive from two different metabolic pathways: from the glucose metabolism through the β-oxidation of acetoacetic acid or from the hydrogenation of isopropanol [17]. At physiological concentrations (133 ppb–6 ppm) [18], acetone is related to the energy metabolism. Conversely, at higher concentration, acetone is considered as a biomarker for diabetes mellitus and type I diabetes [19]. 2-butanone and 2-pentanone are possible biomarkers for lung [11,20] and bladder [21] cancer. In these above-mentioned studies, VOCs (acetone, 2-butanone, and 2-pentanone) are detected using GC–MS analysis after a solid-phase micro-extraction (SPME) [21,22]. With our method, we are able to identify these molecules by reducing the analysis time, which emphasises its potential for clinical studies.

4-heptanone is a common volatile constituent of human urine; it is of unknown origin and it may arise from in vivo decarboxylation of an oxoacid (3-oxo-2-ethylhexanoic acid) from plasticisers with a similar process to acetone from acetoacetic acid [23]. Different research studies, based on headspace solid-phase micro-extraction (HS-SPME) coupled with the GC–MS technique, report 4-heptanone as a possible biomarker for bladder [21], breast [24], lung [11], and renal cell [22] carcinoma.

Among the volatile sulphur compounds, dimethyl sulphide is highly present in urine and is a major contributor to their odour [1]. This VOC is considered as a biomarker for the lung and colorectal cancer [11,25]. To the best of our knowledge, no data have been collected on diallyl sulphide.

Esters are not common urinary VOCs. Among them, only ethyl acetate is shown as a putative biomarker for lung cancer. It has been detected in urine by a headspace GC equipped with a programmed temperature vaporiser and mass spectrometry detector (HS–PTV–GC–MS) [26]. Aldehydes can be produced from the oxygen free-radical-mediated lipid peroxidation of fatty acids. Hexanal is one of the most common aldehydes found in urine [27]. It has been detected with SPME–GC–MS [20,21], Needle Trap Micro-Extraction (NTME) GC–MS [11], and HS–GC–MS [26] and is considered a potential biomarker for many types of cancer such as bladder [21], colorectal [25], leukaemia [16], prostate [28], and especially for lung cancer [29]. Heptanal is the second most found aldehyde in urine samples. In particular, a decrease in its concentration is related to lung [29], colorectal, leukaemia, and lymphoma cancer [16], while an increase in its levels is related to head and neck cancer [30].

The most widely used technique for detecting aldehydes is SPME–GC–MS. A study performed by Khalid et al. identified pentanal as a biomarker for prostate cancer [31]. Propanal is also detected in all our samples, but no other evidence has been collected so far.

Alcohols can have different origins such as the reduction of fatty acids in the gastrointestinal tract [32]. To the best of our knowledge, ethanol, n-propanol, and n-butanol are the most common alcohols in urine and their concentration increases for diabetic patients [33]. Many of the other compounds could be produced by exogenous sources such as food.

Taking into account all the results, although the number of VOCs detectable by other techniques are higher than ours, our method is able to overlap the detection of many compounds. As an example, in the recent study of Taunk et al. [34], the authors showed a volatilomic urinary profile for patients with lung cancer compared to healthy controls using the headspace solid-phase microextraction technique combined with the GC–MS methodology. Interestingly, many VOCs related to clinical differences, such as acetone, 2-butanone, 2-pentanone, 4-heptanone, and toluene, are also detected by our approach.

In parallel, propanal, hexanal, 3-methylbutanal, 2-butanone, and 4-heptanone are widely related to different types of cancer, as reported by Pinto et al.’s study [35]. In addition, Silva et al. [24] described the urinary volatilomic composition of patients with breast cancer and healthy individuals to detect possible VOCs biomarkers. These include some VOCs detectable by our approach, including acetone, 2-butanone, 2-pentanone, hexanal, ethyl acetate, and toluene.

**Figure 4:**
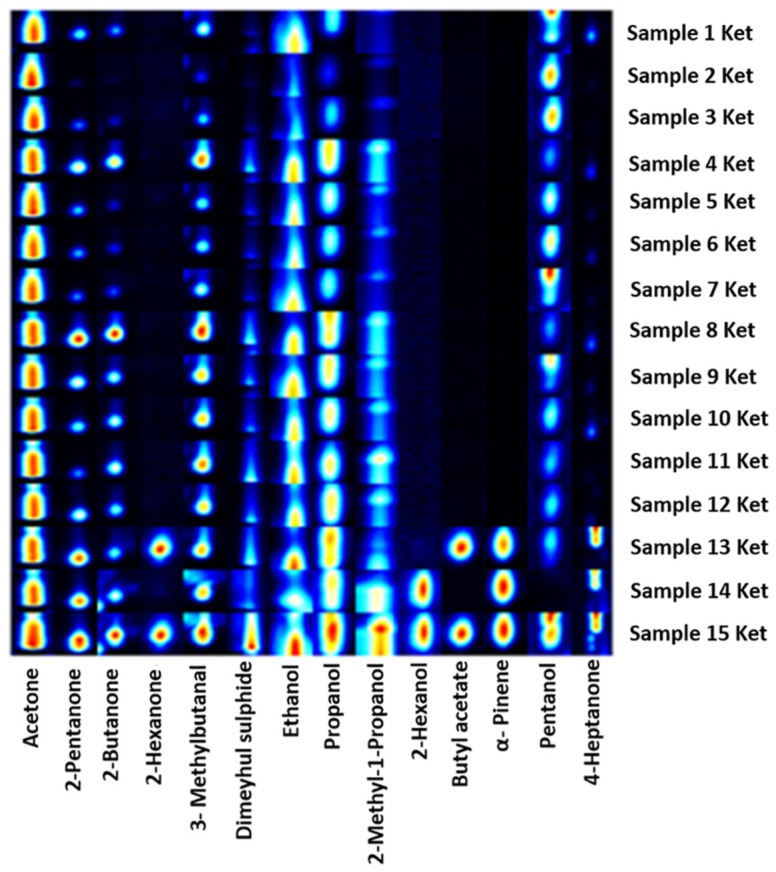
Gallery plot of GC–IMS signals of 14 VOCs species detected in 15 urine samples with ketone bodies value over 60 mg/dL.

Finally, we focus our attention on a specific class of urine samples characterised by an excess of ketone bodies (>60 mg/dL). Compared to the larger population, more alcohols are found in the 15 samples, many of which are present in all of them (Table 4). Among the detected aldehydes, the compounds differ from the rest of the population. With regard to ketones, 2-hexanone is found in addition to the others previously detected and mentioned. Volatile compounds such as acetone, dimethyl sulphide, 3-methylbutanal, propanol, pentanol, and ethanol are found in all our samples as shown in the gallery plot of the main peak areas (Figure 4).

In conclusion, this study aimed to comprehensively profile urinary VOCs by rapid GC–IMS analysis. Based on our results, this methodological approach promises to discriminate VOCs in clinically well-classified patient groups.

We are aware that our study shows some limitations. First, this approach does not allow the quantification of identified VOCs. This would require the development of a more accurate analytical protocol, with the use of specific internal standards, and further investigation of analytical parameters such as precision (repeatability and intermediate precision), limit of quantitation (LOQ), robustness, and recovery [36].

Furthermore, this preliminary study does not take into account contributory factors that may influence both the synthesis and the concentration of VOCs themselves. The latter factors include the clinical features of the population analysed, such as demographic characteristics, diet, alcohol consumption, smoking, and various environmental factors [7,37].

As is well known, the assessment of both pre-analytical and analytical factors is a critical point for the research of biomarkers in biological fluids [38,39,40].

All these important issues, which will be explored in subsequent studies, are beyond the objective of the present manuscript, which, as mentioned, is to obtain a qualitative mapping of the urinary VOCs profile with a rapid screening method.

The overlap of our results with those of other studies mentioned above strengthens the reliability of our proposed method. In this context, GC–IMS stands as a powerful, robust, and easy-to-use technique for separating and detecting VOCs for a rapid, nontargeted screening approach.

## 5. Conclusions

Although GC–MS remains the gold-standard technique for detecting urinary VOCs, it is also extremely time-consuming and expensive. Therefore, it is not a suitable technique to be implemented in the context of fast clinical screening.

With this in mind, we propose an analytically validated alternative method based on the use of GC–IMS for the rapid detection of VOCs in urine, biological fluid widely used in the clinic. This method is not intended to replace more sensitive techniques and must be coupled to analysis for VOCs quantification. However, based on our results, it can represent a first step for rapidly obtaining a profile of urinary VOCs useful for clinical applications.

## Figures and Tables

**Table 1:** Concentration values of ketone mixture standard solutions used for column normalisation and for calibration.

Compound	S_0_ ppm (g/mL)	M_1_ ppb (µg/L)	M_2_ ppb (µg/L)	M_3_ ppb (µg/L)	M_4_ ppb (µg/L)	M_5_ ppb (µg/L)	M_6_ ppb (µg/L)	M_7_ ppb (µg/L)
**2-Butanone**	0.135	216	162	108	70.2	54	27	10.8
**2-Pentanone**	0.135	216	162	108	70.2	54	27	10.8
**2-Hexanone**	0.135	216	162	108	70.2	54	27	10.8
**2-Heptanone**	0.137	218.4	163.8	109.2	71	54	27.3	10.9
**2-Octanone**	0.137	218.4	163.8	109.2	71	54	27.3	10.9
**2-Nonanone**	0.137	218.4	163.8	109.2	71	54	27.3	10.9

Linearity was calculated with standard solutions of 4-heptanone in the range of concentrations of 0–160 ppb, plotting IMS peaks intensity (y-axis) against the 4-heptanone concentration (x-axis). Slope regression was calculated with a linear regression analysis. The minimum concentration value for which an IMS signal is measured, corresponding to the detection limit (limit of detection, LOD), was calculated from the slope regression in order to evaluate the sensitivity of the method.

**Table 2:** Experimental conditions of GC–IMS device. Technical parameters have been schematised both for chromatographic elution (column, carrier gas, flow control, injection volume, and sampling) and ion mobility mass spectrometry (ionisation, model, drift gas, and detector).

GC-IMS Technical Parameters	
Gas Chromatograph	
Column	Capillary, DB wax
Carrier Gas	Air, CGFU Circular Gas Flow Unit
Flow Control	Electronic pressure controller
Injection Volume	3 mL
Sampling	Heated 6-port-valve incl. sample pump
Ion Mobility Spectrometer	
Ionisation	API, ^3^H-Tritium Source (<380 MBq)
Model	Time-of-flight/10 cm tube, ±5000 V
Drift Gas	Air, CGFU Circular Gas Flow Unit
Detection	Faraday Plate

**Table 3:** Summary of VOCs detected across the population in urine samples. ^(a)^ class of molecule to which the VOC belongs; ^(b)^ list of detected VOCs; ^(c)^ retention time (Rt) to which the VOC was eluted; ^(d)^ retention index (Ri) of VOC calculated by the VOCal software; ^(e)^ percentage of the population in which VOCs were detected.

Class ^(a)^	VOCs ^(b)^	Rt [s] ^(c)^	Ri ^(d)^	% ^(e)^
Ketones	Acetone	119	812	100
	2-butanone	141	897	100
	2-pentanone	177	979	97
	4-heptanone	334	1125	16
	2-hexanone	256	1070	0.87
Aldehydes	Propanal	112	763	87
	Pentanal	176	977	44
	Hexanal	255	1070	28
	3-methylbutanal	159	945	13
	Heptanal	385	1152	11
Sulphur compounds	Dimethyl sulphide	107	718	21
	Diallyl sulphide	405	1161	0.87
Alcohols	Ethanol	154	934	100
	Propanol	217	1033	16
	Pentanol	574	1226	13
	2-methyl-1-propanol	271	1083	13
	2-methyl-1-butanol	527	1209	1.74
	2-hexanol	528	1210	0.87
Esters	Butyl acetate	281	1091	72
	Pentyl acetate	338	1153	51
	Ethyl acetate	141	901	16
Aromatic Hydrocarbons	Toluene	228	1045	13
Terpen	α-pinene	175	974	16

**Table 4:** Summary of VOCs detected in urine with excess of ketone bodies. ^(a)^ class of molecule to which the VOC belongs; ^(b)^ list of detected VOCs; ^(c)^ number of samples that contain the VOC; ^(d)^ putative origin of the detected VOCs (Endo = VOC endogenously produced; Exo = VOC resulting from exogenous sources (food, environment, an medication); M = VOC from microbial metabolism; D = VOC from drug metabolism, as reported by Porto-Figueira et al. [11]).

Class ^(a)^	VOCs ^(b)^	Number of Samples ^(c)^	Origin ^(d)^ [11]
Ketones	Acetone	15	Endo, M
	2-pentanone	14	Exo (Food)
	2-butanone	12	Endo
	4-Heptanone	7	Endo
	2-hexanone	1	Endo
Aldehydes	3-methylbutanal	15	Unknown
Sulphur compounds	Dimethyl sulphide	15	Endo/Exo (D, M)
Alcohols	Ethanol	15	Unknown
	Propanol	15	Unknown
	Pentanol	15	Unknown
	2-methyl-1-propanol	15	Unknown
	2-hexanol	1	Unknown
Esters	Butyl acetate	2	Unknown
Terpenes	α-pinene	3	Endo/Exo (Food)

## Data Availability

The data presented in this study are available in article.
